# Experimental Study on Enhancing the Mechanical Properties of Sandy Soil by Combining Microbial Mineralization Technology with Silty Soil

**DOI:** 10.3390/ma17102362

**Published:** 2024-05-15

**Authors:** Jun Hu, Fei Fan, Luyan Huang, Junchao Yu

**Affiliations:** 1 School of Civil Engineering and Architecture, Hainan University, Haikou 570228, China; hj7140477@hainanu.edu.cn (J.H.); fanfei_hn@163.com (F.F.); 22220856000069@hainanu.edu.cn (L.H.); 2 Collaborative Innovation Center of Marine Science and Technology, Hainan University, Haikou 570228, China; 3 School of Architecture and Art Design, Nanjing Polytechnic Institute, Nanjing 210048, China

**Keywords:** MICP, silty soil, triaxial shear test, calcium carbonate generation rate, SEM

## Abstract

Currently, coastal sandy soils face issues such as insufficient foundation strength, which has become one of the crucial factors constraining urban development. Geotechnical engineering, as a traditional discipline, breaks down disciplinary barriers, promotes interdisciplinary integration, and realizes the green ecological and low-carbon development of geotechnical engineering, which is highly important. Based on the “dual carbon” concept advocating a green and environmentally friendly lifestyle, Bacillus spores were utilized to induce calcium carbonate precipitation technology (MICP) to solidify coastal sandy soils, leveraging the rough-surface and low-permeability characteristics of silty soil. The mechanical-strength variations in the samples were explored through experiments, such as calcium carbonate generation rate tests, non-consolidated undrained triaxial shear tests, and scanning electron microscopy (SEM) experiments, to investigate the MICP solidification mechanism. The results indicate that by incorporating silty soil into sandy soil for MICP solidification, the calcium carbonate generation rates of the samples were significantly increased. With the increase in the silty-soil content, the enhancement range was 0.58–3.62%, with the maximum calcium carbonate generation rate occurring at a 5% content level. As the silty-soil content gradually increased from 1% to 5%, the peak deviator stress increased by 4.2–43.2%, enhancing the sample shear strength. Furthermore, the relationship between the internal-friction angle, cohesion, and shear strength further validates the enhancement of the shear strength. Silty soil plays roles in adsorption and physical filling during the MICP solidification process, reducing the inter-particle pores in sandy soil, increasing the compactness, providing adsorption sites, and enhancing the calcium carbonate generation rate, thereby improving the shear strength. The research findings can provide guidance for reinforcing poor coastal sandy-soil foundations in various regions.

## 1. Introduction

With the rapid development of coastal cities, land resources are being consumed at a fast pace. However, many sandy-soil foundations lack sufficient strength, which has become one of the key factors constraining urban development. Currently, in many coastal areas, phenomena such as the displacement and breakage of engineering piles have occurred [1]. Therefore, the effective reinforcement of sandy-soil foundations is essential in the construction planning of coastal cities to address the challenges posed by poor geotechnical engineering conditions.

For the treatment of poor sandy-soil foundations, the traditional methods generally use the replacement of bedding, cementation reinforcement, reinforcement treatment, drainage reinforcement, etc. [2]. Although these methods can improve the bearing capacity of the soil to a certain extent, there will be noise, construction difficulties, harmful gases, and other shortcomings. In recent years, MICP technology has been recognized as an emerging low-carbon and environmentally friendly microbial reinforcement technology [3,4], and it has been widely used to improve the soil stability [5,6], cure sandy soils [7,8,9], repair concrete cracks [10,11], treat contaminated soils [12,13], and enhance the erosion resistance of slopes [14,15]. According to the different types of metabolism, the main reaction mechanisms of MICP technology can be divided into the urea hydrolysis reaction [16,17,18], the denitrification process [19,20], the sulfur reduction process [21], the iron reduction process [22,23], and so on. In geotechnical engineering, the most researched is the use of the urea hydrolysis reaction of the MICP process. The microorganisms selected for this process are generally those of the high-urease-producing Bartonella species, due to its environmental adaptability, and those of the high-urease-producing Bacillus subtilis, which has strong environmental adaptability and high activity and exists in large quantities in the soil. This technology can produce a kind of urease through the physiological metabolism of Bacillus subtilis, which can decompose urea to produce NH_4_^+^ and CO_3_^2−^. Moreover, there are negative charges and adhesins on the surface of the cell wall of Bacillus subtilis, so that it can adsorb the calcium ions in the cementation liquid to generate calcium carbonate, which can cement the geotechnical particles as a whole with the mechanical strength. The main reaction chemical formulae involved are as follows [24,25]:

Ca^2+^ + Cell → Cell-Ca^2+^ (1)

NH_2_-CO-NH_2_ + 2H_2_O → 2NH_4_^+^ + CO_3_^2−^ (2)

CO_3_^2−^ + Cell-Ca^2+^ → Cell-CaCO_3_ (3)

In the pursuit of enhancing the reinforcement efficacy of Microbially Induced Calcium Carbonate Precipitation (MICP) techniques, numerous scholars have introduced auxiliary materials into the research framework to augment the strength or stiffness during the MICP reinforcement process. For instance, Kumar et al. [26] observed a significant increase in the strength under the unconfined strength compression (USC) test when the soil contained substantial clay contents. Ramachandran et al. [27] noted that incorporating xanthan gum into soil improves roadbed stability. Nikseresht et al. [28] demonstrated that the addition of sugar cane molasses and vinasse enhances calcium carbonate production. Zhao et al. [29] amalgamated sand with varying proportions of activated carbon for reinforcement, resulting in heightened unconfined compressive strengths and bacterial retention rates of the sand samples. Additionally, Xie et al. [30], Zheng et al. [31], and Xiao et al. [32] advocated for the integration of fibers into sandy soils to bolster the strength and stiffness of reinforced specimens. Zhang Guocheng [33] and Ma Ruinan et al. [34] observed that a small quantity of Mg^2+^ could moderately enhance the compressive strength of cured sand columns. Zhi Yongyan [35] demonstrated that the addition of the chemical reagents NH_4_Cl and NaHCO_3_ could elevate calcium carbonate precipitation. While the incorporation of auxiliary materials can augment the mechanical strength and stiffness of specimens, it often entails high costs. In this study, we leveraged waste silty soil, abundantly available in coastal regions, for its practical convenience in local sourcing and reduced transportation expenses. Our aim is to achieve the objective of waste remediation using waste materials. Silty soil inherently possesses low permeability and surface roughness. By combining MICP technology with the modification and curing of sandy soil using silty soil, we anticipate alterations in the calcium carbonate generation rate and mechanical strength.

In order to investigate the effect of silty soil combined with MICP technology on the bearing capacity of sandy-soil foundations in coastal areas, we synthesized the ideas in the existing literature by mixing small-size silty soil into sandy soil to improve the degree of the densification of the specimen, reinforced it with MICP technology, and investigated the mechanical properties of the cured specimens through the calcium carbonate generation rate test and unconsolidated and undrained triaxial shear test, as well as the mechanism of its strength enhancement through SEM tests. The results of the study can provide guidance for the reinforcement of poor coastal sandy-soil foundations in various regions.

## 2. Experimental Materials and Methods

### 2.1. Test Materials

**Figure 1:**
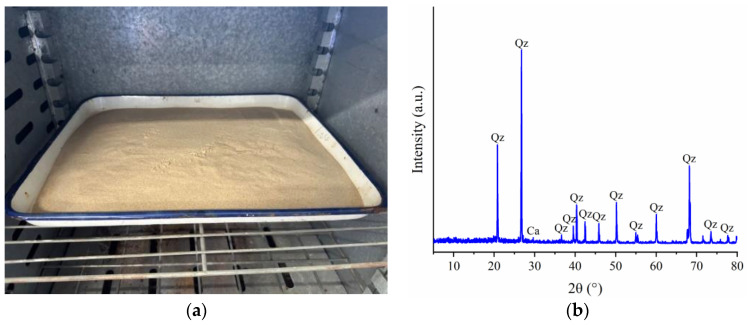
Sandy soil in Jiangdong New Area, Haikou City: (**a**) sandy soil from Jiangdong New Area; (**b**) X-ray diffraction (XRD) analysis (Qz, quartz; Ca, calcite).

The experimental sand used from Jiangdong New Area, Haikou City, Hainan Province, as shown in Figure 1a [36], has a particle size distribution mainly ranging from 0.25 mm to 0.5 mm. The particles are yellowish-brown, with a smooth surface. X-ray diffraction (XRD) analysis of the samples after triaxial shear failure using a Bruker D8 ADVANCE instrument (Ettlingen, Germany), as shown in Figure 1b [37], revealed that its composition is predominantly SiO_2_, accounting for 97.6% by mass, with a small amount of CaCO_3_, accounting for 2.4% by mass.

**Figure 2:**
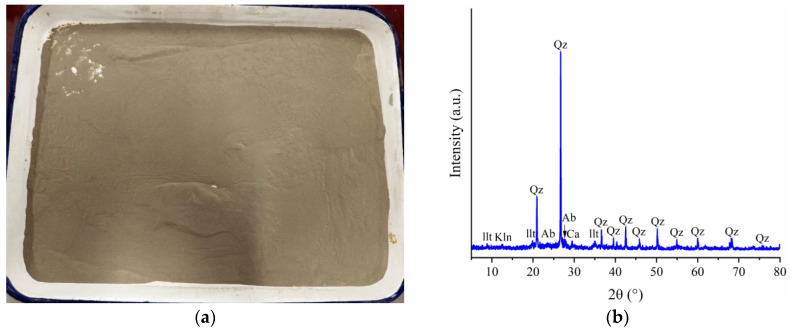
Silty soil in Jiangdong New Area, Haikou City, China: (**a**) silty soil; (**b**) X-ray diffraction (XRD) analysis. (llt, illite; Kln, kaolinite; Qz, quartz; Ab, albite; Ca, calcite).

The experimental silty clay was obtained from Jiangdong New Area, Haikou City [36], with a particle size range from 0 mm to 0.25 mm after crushing, as shown in Figure 2a, which is smaller than the particle size of sand. It contains abundant metal compounds, such as Na, K, and Al, and has complex organic matter. Its permeability coefficient is in the range from 10^−5^ to 10^−7^ cm/s. X-ray diffraction (XRD) analysis, as shown in Figure 2b, revealed the following composition by mass percentage: SiO_2_ (54.1%), CaCO_3_ (2.3%), NaAlSi_3_O_8_ (5.9%), Al_2_(Si_2_O_5_)(OH)_4_ (6.9%), and kaolinite (Al_2_Si_2_O_5_(OH)_4_) (30.8%).

### 2.2. Test Broths and Media

The bacterial strain used in this experiment was Bacillus subtilis (American Type Culture Collection strain number ATCC 11859), purchased from the Guangdong Culture Collection Center (Guangzhou, China). The strain was cultured on slants, with a slightly curved morphology and dimensions of 0.5–0.8 × 5.4 × 12.2 µm. This strain is well known for its high activity and strong ability to produce calcium carbonate in the MICP process, with clear reaction mechanisms. The culture medium used was a yeast liquid medium, and the composition per liter is shown in Table 1 [38]. Following inoculation at a ratio of 1:50 (bacterial solution to culture medium), the culture was incubated on a constant temperature shaker at 30 °C and 190 rpm for 48 h to achieve maximum activity under the experimental conditions, with a maximum velocity of 0.466 ms/min and an optical density (OD_600_) of 2.488.

### 2.3. Sample Preparation

**Figure 3:**
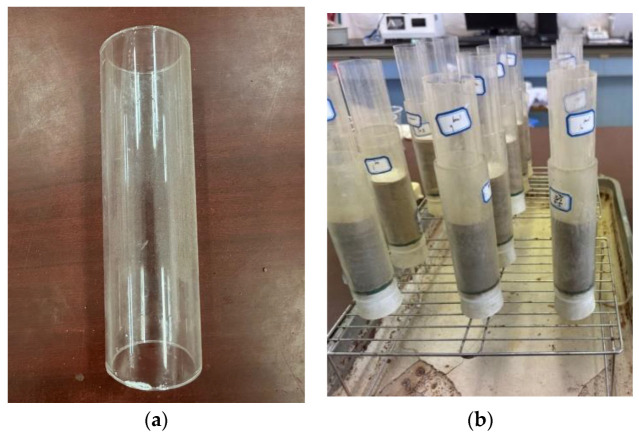
Making a standard specimen: (**a**) acrylic mold; (**b**) post-production.

In this experiment, cylindrical, transparent molds made of acrylic material with an inner diameter of 39.1 mm, a height of 130 mm, and a wall thickness of 3 mm (as shown in Figure 3a) were used. These molds facilitated the observation of the permeation of the bacterial solution and cementing solution within the samples. The sample dimensions were set to φ39.1 mm × 80 mm. To facilitate subsequent demolding, a layer of PVC film was placed between the sample and the mold, and Vaseline was evenly applied. To investigate the effect of silty soil on the mechanical strength of sand after MICP solidification, samples were prepared by uniformly mixing 1%, 2%, 3%, 4%, and 5% of silty soil into the sand. To ensure the uniformity of the samples and prevent fracture after solidification, a sample preparation method similar to that described by XIAO Y et al. [39] was followed. The sand was added to the mold in three increments, with compaction and brushing between each addition. A rubber plug with holes was used at the bottom of the mold. These holes allowed excess bacterial solution and cementing solution to flow out from below during the permeation process after adsorption and reaction in the sample pores. Between the rubber plug and the sand, a layer of gauze and filter paper was placed to prevent sand particles from leaking out through the holes in the rubber plug. After the sample was prepared, a layer of filter paper was placed on the top of the sample to prevent uneven surfaces caused by the impact of the bacterial solution during grouting, as shown in Figure 3b.

### 2.4. Curing Process

**Figure 4:**
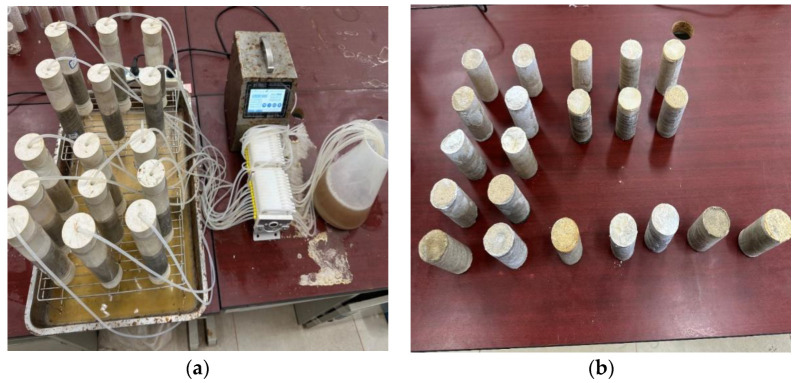
Grouting sampling process: (**a**) peristaltic pump grouting; (**b**) specimen after drying.

During the solidification process of the samples, a peristaltic pump was initially utilized to administer the bacterial solution to the samples at a controlled rate of 3 mm/min, as depicted in Figure 4a. Following a 12 h period of standing, a cementing solution comprising a 1:1 mixture of 0.5 mol/L urea and 0.5 mol/L calcium chloride was subsequently introduced into the samples. This injection process was reiterated 3–5 times until saturation was achieved, indicated by the inability of the liquid to permeate through the sample. Subsequent to demolding, the samples underwent a thorough rinsing with water to eliminate any residual chemicals from the interior. Given the surface irregularities resulting from the injection process, the upper and lower surfaces of the samples were meticulously leveled using a file. The dried samples are depicted in Figure 4b.

### 2.5. Calcium Carbonate Generation Rate

Using the acid-washing method to measure the rate of calcium carbonate formation [40], the mass of the sample after it was solidified using MICP technology, rinsed with water, and dried is denoted as *M*_1_. After soaking in excess hydrochloric acid for 24 h, rinsing with excess water, and drying, the mass of the sample is denoted as *M*_2_. The rate of calcium carbonate formation is denoted as *M*.

(4) $$M=\frac{M_{1}-M_{2}}{M_{2}}\times 100\%$$

**Figure 5:**
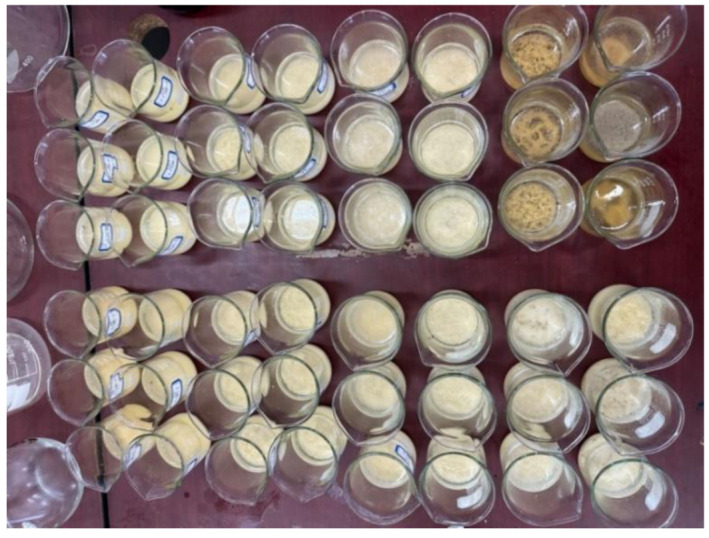
Calcium carbonate generation rate test.

To investigate the distribution of calcium carbonate within the samples, the solidified samples were divided uniformly into three sections: top, middle, and bottom, after which acid-washing experiments were conducted, as shown in Figure 5.

### 2.6. Triaxial Shear Test

**Figure 6:**
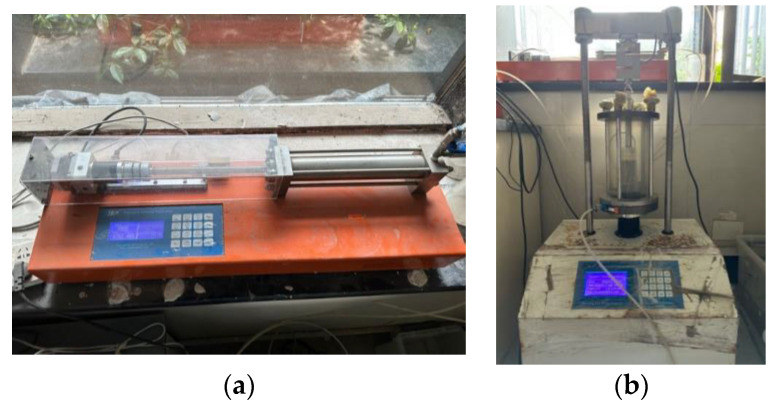
Triaxial shear test apparatus: (**a**) confining-pressure controller; (**b**) pressure chamber.

The triaxial shear test is an important method for obtaining the engineering mechanical parameters of sand indoors and is a method for determining the shear strength of sand. The strength parameters obtained from the triaxial shear test were more representative of the mechanical parameters of the sand used in outdoor MICP solidification in this experiment. To meet the experimental principles and enhance the accuracy of the shear strength indicators, four specimens were used under different proportions of silty clay. Shear failure was considered under effective confining pressures of 100 kPa, 200 kPa, 300 kPa, and 400 kPa [41]. The instruments used are shown in Figure 6.

The steps of the triaxial shear test are as follows:

(1) Before conducting the test, check for any damage to the rubber membrane, ensure the smoothness of all inlet and outlet pipelines, and purge any air bubbles in the pore water pressure system;
(2) Use a membrane carrier to wrap the rubber membrane around the sample. Use an ear syringe to remove excess air inside the rubber membrane. Place the prepared sample into the membrane carrier;
(3) Place pervious stones at the bottom of the pressure chamber, then place the sample on top of the pervious stones. Remove the support cylinder and place pervious stones and the sleeve on top of the sample. Securely tie the ends of the rubber membrane and install the pressure-type outer casing;
(4) After tightening the outer-casing screws, open the inlet valve and fill the pressure chamber with water. Close the inlet valve after the pressure chamber is filled with water, apply the designed confining pressure, and load at a rate of 1 mm/min;
(5) Stop the test when the axial strain reaches 15%. Take the difference in the principal stresses at 15% axial strain as the failure point and calculate the UU test strength parameters based on the Mohr–Coulomb criterion. Afterward, unload the confining pressure, remove the outer-casing pressure, and record the data, such as the shear stress–strain.

### 2.7. SEM Curing Mechanism Analysis

**Figure 7:**
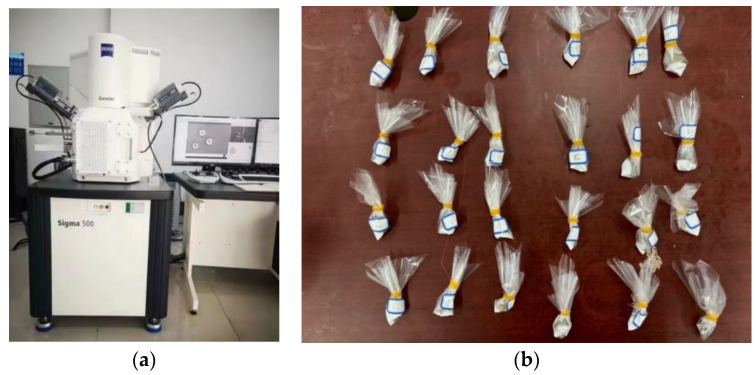
Test apparatus and test samples: (**a**) Sigma 500 scanning electron microscope; (**b**) test samples.

To delve into the mechanism underlying the incorporation of silty soil into coastal sandy soil post-treatment with Microbially Induced Calcium Carbonate Precipitation (MICP) technology, microscopic analysis via scanning electron microscopy (SEM) was employed to investigate the factors influencing the strength variation in the specimens. A combination of macroscopic and microscopic approaches was adopted to elucidate the role of silty soil in the solidification process and its impact on the mechanical strength of the specimens. The experimental setup utilized the Sigma 500 model SEM (ZEISS, Jena, Germany), as depicted in Figure 7a, with specimens sourced from the triaxial shear experiments following specimen destruction, as illustrated in Figure 7b. SEM analysis was conducted at varying magnifications, including 100×, 200×, 500×, 1000×, and 5000×, to scrutinize the internal reinforcement within each segment of the specimen. This comprehensive analysis aimed to ascertain the influence of mixing silt loam on the specimen’s response to MICP curing.

## 3. Results and Discussion

### 3.1. Calcium Carbonate Generation Rate Test

**Figure 8:**
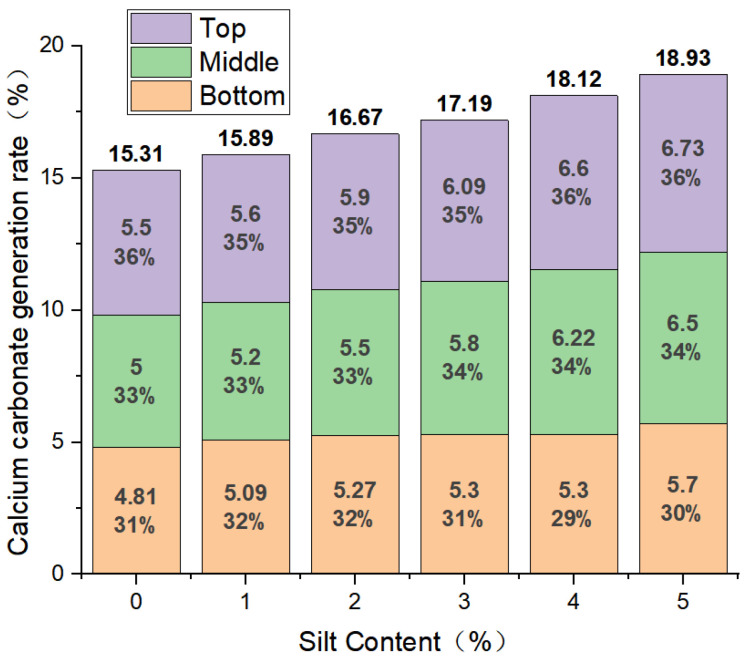
Calcium carbonate generation rates at the upper, middle, and lower sections of the specimens with different proportions of silty clay.

According to the variations in the contents of silty soil, the calcium carbonate generation rates of the top, middle, and bottom sections of the samples reinforced using MICP technology were plotted for different silty-soil content levels, as shown in Figure 8 [42,43].

Prior to the implementation of Microbially Induced Calcium Carbonate Precipitation (MICP) technology for solidification, the calcium carbonate content within the loose sand particles was negligible. Following solidification, there was a notable augmentation in the internal calcium carbonate contents of the samples. The rates of calcium carbonate generation exhibited variability contingent upon the silty-soil content. Relative to the samples devoid of silty soil, the calcium carbonate generation rates escalated by 0.58%, 1.36%, 1.88%, 2.81%, and 3.62% as the silty-soil contents increased from 1% to 5%. Throughout the experiments, the proportions of calcium carbonate generated in the lower segments of individual samples were relatively diminished compared to those of the middle and upper segments. Generally, the calcium carbonate generation rates in the middle segments were either equivalent to or less than those in the upper segments. Despite the fluctuations in the silty-soil content, there were no discernible alterations in the proportions of the calcium carbonate generation rates among the upper, middle, and lower segments of the samples. Consequently, the incorporation of silty soil into the sand particles amplified the calcium carbonate generation rates of the samples under MICP technology, with the maximum rate achieved at a silt soil content of 5%. Nevertheless, the enhancement in the uniform distribution of calcium carbonate within the samples was marginal.

### 3.2. Unconsolidated and Undrained Triaxial Shear Test

#### 3.2.1. Mechanical-Strength Properties

**Figure 9:**
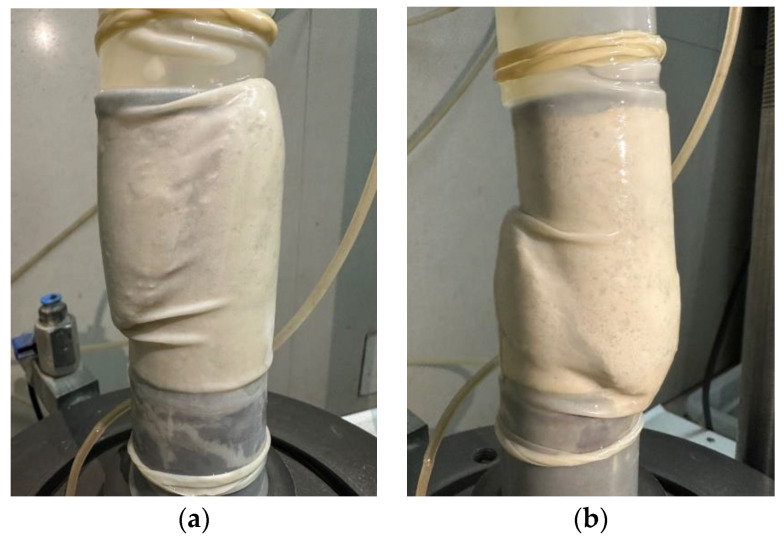
Triaxial shear failure specimen: (**a**) shear damage; (**b**) shear damage + dropsy damage.

In the different silty-soil mixes, the loose sand particles from the specimens cemented into a certain strength in the sand columns. After the triaxial shear test, shear cracking occurred, as seen in a single cross section of the shear damage in Figure 9, but, due to the MICP-reinforcing technology, the loose sand particles were still inhomogeneous, so the bottom of the specimen may have been due to dropsy damage, as shown in Figure 9b.

**Figure 10:**
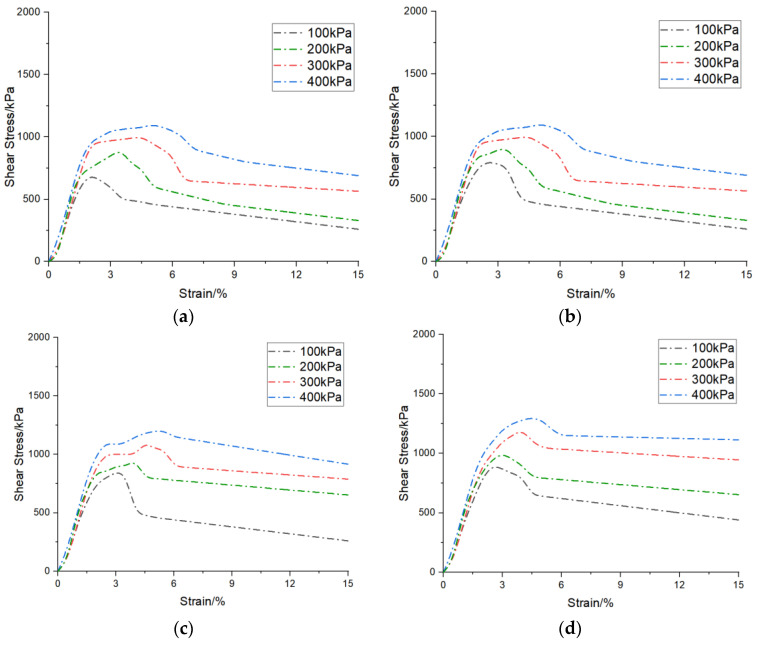

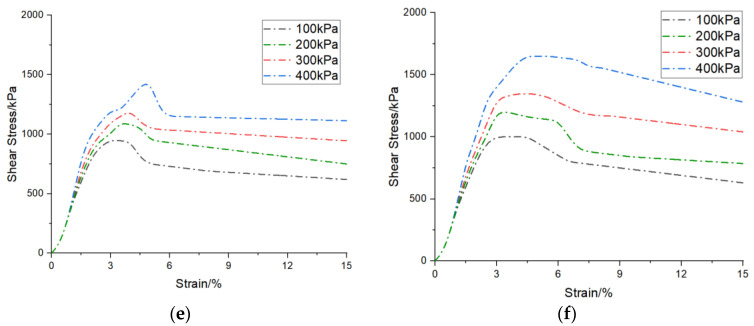
Bias stress–strain curves for triaxial shear test: (**a**) silty soil = 0%; (**b**) silty soil = 1%; (**c**) silty soil = 2%; (**d**) silty soil = 3%; (**e**) silty soil = 4%; (**f**) silty soil = 5%.

In order to investigate the mechanical strengths of the specimens under triaxial shear test, the bias stress–strain curves were derived from the compressive shear test at four peripheral pressures, 100 kPa, 200 kPa, 300 kPa, and 400 kPa, and they are shown in Figure 10.

Across various confining pressures, the deviator stress initially experienced exponential growth with the increasing strain, followed by a subsequent ascent before declining and ultimately tapering off after the deviator stress reduction, thereby manifesting a strain-softening phenomenon. An examination of the deviator stress–strain curves identifies the peak deviator stress as the failure threshold, with the corresponding strain at peak deviator stress delineating the peak strain. Notably, for a given sample subjected to a confining pressure of 400 kPa, the peak deviator stress exceeded those observed under 300 kPa, 200 kPa, and 100 kPa. Concurrently, as the confining pressure escalated, both the peak deviator stress and residual strength demonstrated consistent augmentation. Furthermore, the residual strength consistently registered lower values under low confining pressures compared to under high confining pressures. Additionally, the strain at which the sample attained peak stress under low confining pressure proved to be smaller than that under high confining pressure.

Prior to the introduction of silty soil, the deviator stress–strain profiles of the reinforced specimens exhibited analogous variations, typically delineated into distinct stages: the elastic, yielding, and residual-stress stages [44]. Preceding the attainment of peak strength, the deviator stress incrementally rose with the axial strain, with no conspicuous disparity observed in the gradient of the curve. This observation suggests that the incorporation of silty soil exerted minimal influence on the initial stiffness of the samples treated with Microbially Induced Calcium Carbonate Precipitation (MICP). Nevertheless, subsequent to the inclusion of silty soil, the peak deviator stresses of the samples surpassed those of the pure-sand specimens, signifying an enhancement in the soil’s load-bearing capacity. For pure-sand specimens subjected to confining pressures of 100 kPa, 200 kPa, 300 kPa, and 400 kPa, the peak deviator stresses were recorded as 713 kPa, 895 kPa, 1032 kPa, and 1152 kPa, respectively. In comparison to the pure-sand specimens, with the gradual escalation in the silty-soil content from 1% to 5% under confining pressures of 100 kPa, 200 kPa, 300 kPa, and 400 kPa, the peak deviator stresses exhibited increments ranging from 4.2% to 30.8%, from 6.1% to 33.3%, from 6.6% to 42%, and from 12.2% to 43.2%, respectively [41].

At low confining pressures, the strain associated with the peak deviator stress was comparatively modest. As the confining pressure rose, the strain corresponding to the peak deviator stress of the specimens also escalated, signifying that the specimens treated with Microbially Induced Calcium Carbonate Precipitation (MICP) exhibited commendable ductility under elevated confining pressures. However, the peak strains of the specimens under confining pressures ranging from 100 kPa to 400 kPa exhibited no discernible trend with the increase in the silty-soil content, suggesting that the addition of silty soil exerted a negligible influence on the peak strain of the MICP-treated specimens.

#### 3.2.2. Shear Strength Index Analysis

The shear strength of cohesive soil denotes the utmost shear stress it can endure prior to experiencing shear failure, which is typically characterized by the cohesion and internal-friction angle. To examine the alterations in the shear strength of the reinforced sandy soil, a Mohr stress circle was constructed utilizing the extracted stress–strain relationship data, enabling the determination of the cohesion and internal-friction angle for the reinforced soil via tangent-line drawings. The investigation delved into the fluctuations in the shear strength observed in the specimens before and after the incorporation of silty soil via Microbially Induced Calcium Carbonate Precipitation (MICP) technology for solidification purposes.

During the undrained triaxial shear test, the peak strength was determined based on the experimental data. Next, within the plane marked with shear stress on the vertical axis and normal stress on the horizontal axis, the failure stress circles were plotted for confining pressures of 100 kPa, 200 kPa, 300 kPa, and 400 kPa, with the center as $\;\frac{\left(\sigma 1+\sigma 3\right)}{2}$ at failure and $\frac{\left(\sigma 1-\sigma 3\right)}{2}$ as the radius [41]. Strength envelopes were also plotted. Through these graphs, we obtained the shear strength parameters. In these graphs, the intercept of the envelope on the vertical axis represents cohesion (c), and the angle with the horizontal axis represents the internal-friction angle (φ). Calculations were performed based on the Mohr–Coulomb failure criterion [45]:

(5) $$\sigma_{1}-\sigma_{3}=\frac{2}{1-sin\varphi }(ccos\varphi -\sigma_{3}sin\varphi )$$

In the formula, ($\sigma_{1}-\sigma_{3}$) represents the difference in the principal stresses at failure; $\sigma_{3}$ represents the confining pressure applied to the specimen.

The equation above can be simplified as follows:

(6) $$\frac{\sigma_{1}-\sigma_{3}}{2}=ccos\varphi +\frac{\sigma_{1}+\sigma_{3}}{2}sin\varphi$$

**Figure 11:**
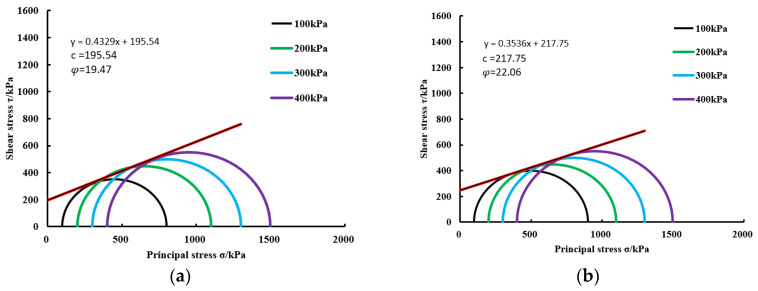

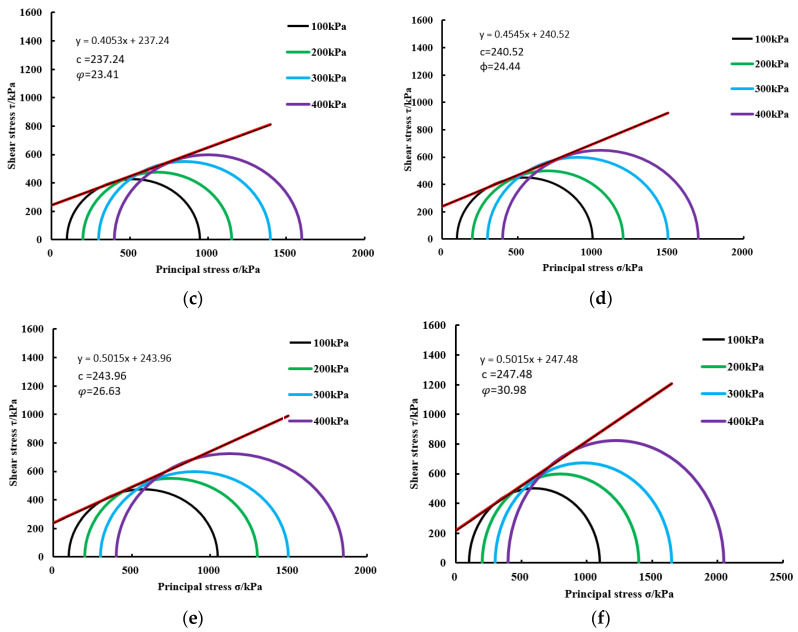
Shear strength envelopes: (**a**) silty soil = 0%; (**b**) silty soil = 1%; (**c**) silty soil = 2%; (**d**) silty soil = 3%; (**e**) silty soil = 4%; (**f**) silty soil = 5%.

The shear strength envelopes, internal-friction angles, and cohesion of each group of specimens are shown in Figure 11.

According to Figure 11, as the confining pressure increased from 100 kPa to 400 kPa, there was a gradual augmentation in the effective stress exerted on the specimens. Concurrently, the diameter of the stress circles pertaining to the specimens exhibited progressive enlargement. After reinforcement employing Microbially Induced Calcium Carbonate Precipitation (MICP) technology, the cohesion of the pure-sand specimens measured 195.54 kPa. Upon introducing the silty-soil admixture at concentrations of 1%, 2%, 3%, 4%, and 5%, the cohesion registered increments of 11.36%, 21.33%, 23%, 24.76%, and 26.56%, respectively. Similarly, the internal-friction angle of the pure-sand specimens stood at 19.47°, with the subsequent introduction of the silty-soil admixture at concentrations 1%, 2%, 3%, 4%, and 5% leading to increases of 13.3%, 20.24%, 25.53%, 36.77%, and 59.12%, respectively. Notably, with the inclusion of silty soil, both the cohesion and internal-friction angles demonstrated ascending trajectories, attaining maximal values at a silty-soil content of 5%. Further substantiating this enhancement, the cohesion and internal-friction angles as indicated by the shear strength envelopes underscore that the incorporation of silty soil into sandy soil for MICP reinforcement markedly enhanced the mechanical robustness of the specimens.

### 3.3. SEM Solidification Mechanism Analysis

**Figure 12:**
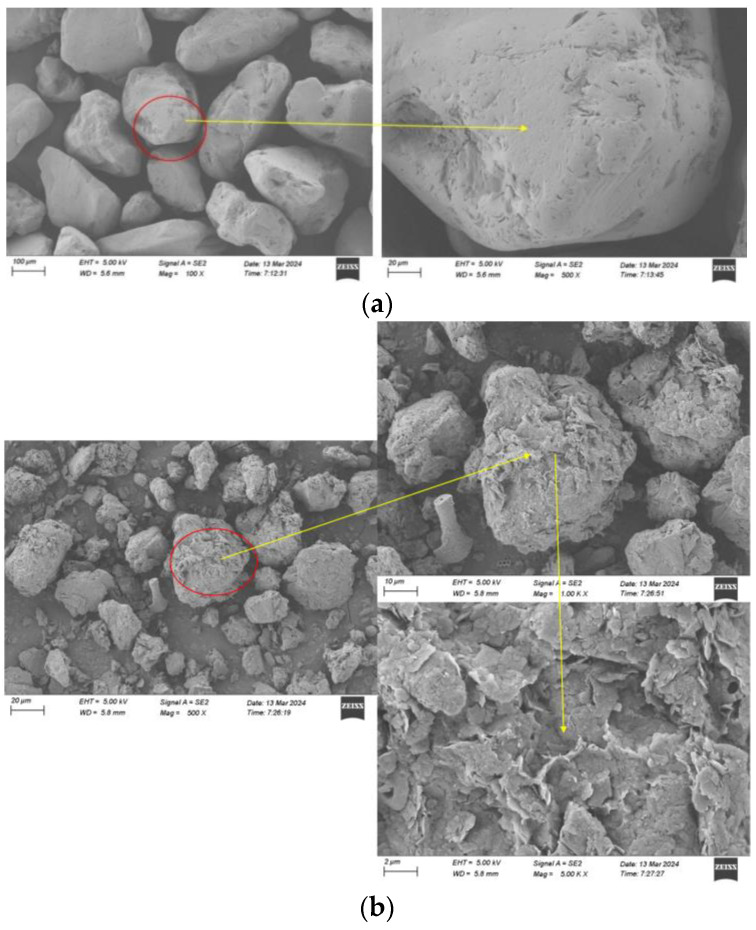
Scanning electron microscopy (SEM) micrographs: (**a**) sandy soil from Jiangdong New Area; (**b**) silty soil.

The enlarged surfaces of sandy- and silty-soil particles under the scanning electron microscope (SEM) are shown in Figure 12. The surfaces of the sand particles appear relatively smooth with fewer pores, shallow grooves, more edges, larger inter-particle voids, and irregular particle distribution. Based on the roughness of their surfaces and the size of their pores, it can be inferred that during the MICP solidification grouting experiment, there might have been fewer adsorption sites for the bacterial solution during the infiltration process, possibly resulting in a lower bacterial adhesion rate.

**Figure 13:**
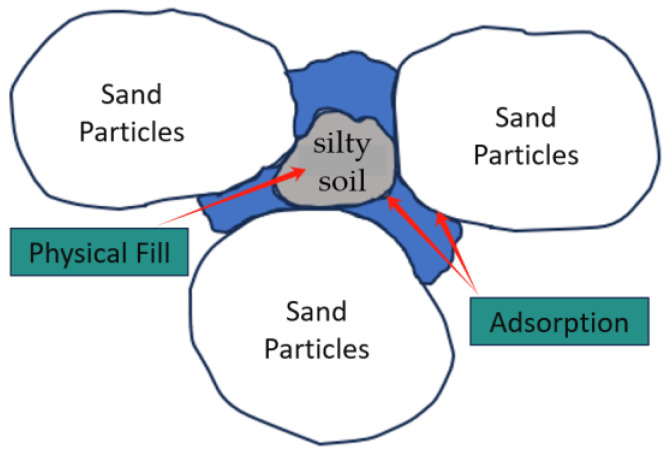
Schematic diagram of adsorption of silty-soil filling.

The silty-soil particles have a smaller diameter than that of the sand particles and possess low permeability. They contain various organic matters. From the microscopic image, it is evident that the particles of this soil are irregular in shape, with numerous protrusions and grooves on the surfaces, and they exhibit irregular particle distribution. Under a magnification of 500 times, the surface appears to have a laminar shell-like structure. Based on the roughness of the surface and particle size, it can be inferred that incorporating silty soil into sandy soil in coastal areas can effectively fill the inter-particle voids, as depicted in Figure 13. Moreover, during grouting, it can provide adsorption sites, thereby enhancing bacterial adhesion.

**Figure 14:**
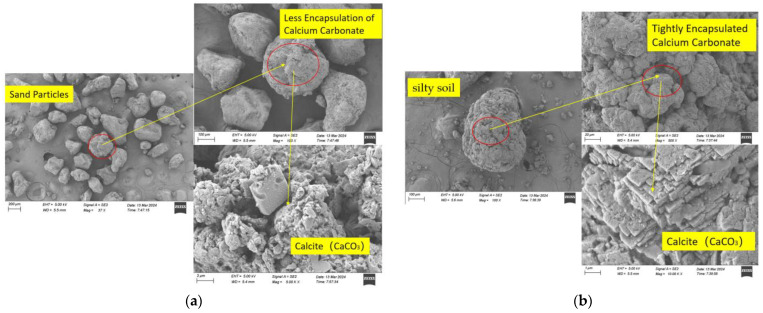
Calcium carbonate encapsulation before and after incorporation of silty soil: (**a**) pure-sand MICP-cured particle surface; (**b**) surface of particle cured by MICP with silty soil incorporated.

When comparing the MICP-solidified specimens incorporating silty clay with those comprising pure sand, there is a notable disparity in the abundance of calcium carbonate crystals present on the surfaces post-solidification, as seen in Figure 14a. This variance is attributed to the smoother surface texture of sand particles, which inherently possess fewer adsorption sites. Despite undergoing multiple grouting cycles, the deposition of calcium carbonate on the surfaces of the sand particles remained limited, thereby leading to a reduction in the mechanical strength of the specimens. In contrast, the silty clay exhibited a higher density of tightly encapsulated calcium carbonate particles on its surface subsequent to solidification, as depicted in Figure 14b. This phenomenon arose from the introduction of silty clay, which effectively filled the voids within the specimens, thereby enhancing their impermeability. Furthermore, the augmented presence of bacterial adsorption sites facilitated by the incorporation of silty clay facilitated a gradual escalation in calcium carbonate generation. Consequently, a positive correlation emerged between the quantity of the silty clay incorporated and the amount of calcium carbonate produced. With the progressive increase in calcium carbonate production, the internal cohesion within the specimens intensified, consequently bolstering the mechanical strength under triaxial shear testing.

## 4. Conclusions

In order to investigate the effect of silty soil combined with MICP technology on the bearing capacity of sandy-soil foundations in coastal areas, silty soil with a small grain size was mixed into sandy soil and reinforced using MICP technology. The mechanical properties of the cured specimens were investigated by the calcium carbonate generation rate test and unconsolidated undrained triaxial shear test, and the strength improvement mechanism was explored by the scanning electron microscopy (SEM) test. The main conclusions are as follows:

(1) Compared with the pure-sand specimens, the calcium carbonate generation rates increased from 15.31% to 18.93% with the addition of 1–5% silty clay, indicating that the addition of silty clay during the experiment increased the calcium carbonate generation rates of the specimens. The cementitious material increased with the increase in the amount of silty clay added, reaching the optimum dosage at 5%;
(2) The shear strength curve shows the strain-softening phenomenon. As the content of silty clay increased from 1% to 5%, the peak deviator stress increased by 4.2–43.2%, indicating that the addition of silty clay enhanced the shear strength of the specimens. The strength index cohesion and internal-friction angles were further verified through the shear strength envelope curves;
(3) SEM experiments showed that the strengthening mechanism of the silty clay on the MICP-solidified sandy soil mainly included the physical-filling and adsorption effects of the particles. After adding silty clay, the overall pore structure of the specimens was optimized, the calcium carbonate generation rate increased, and the cohesion between the particles became stronger, thereby improving the overall strength of the specimens;
(4) In addition, future research could consider simultaneously adding silty clay and fibers to sandy soil to explore whether both the strength and stiffness can be improved. Moreover, future studies could consider changing the concentration of the cementing solution to observe whether lower concentrations can enhance the mechanical strength of MICP specimens with added silty clay.

## Figures and Tables

**Table 1:** Yeast liquid media.

Yeast Culture Medium	Weight	Unit	Brand
Yeast extract	20	g	OXOID
NH_4_Cl	10	g	Shanghai Pharmaceutical Trial
NiCl_2_	24	mg	Damao
MnSO_4_	10	mg	Shanghai Pharmaceutical Trial
H_2_O	1	L	/
NaOH	1	mol/L	XNK

## Data Availability

Data are contained within the article.
